# Supplementary figures and images for: High-throughput and affordable genome-wide methylation profiling of circulating cell-free DNA by methylated DNA sequencing (MeD-seq) of LpnPI digested fragments
Source: Clin Epigenetics. 2021 Oct 20;13:196. doi: 10.1186/s13148-021-01177-4 (PMC8529776; doi:10.1186/s13148-021-01177-4)

## Slide 1
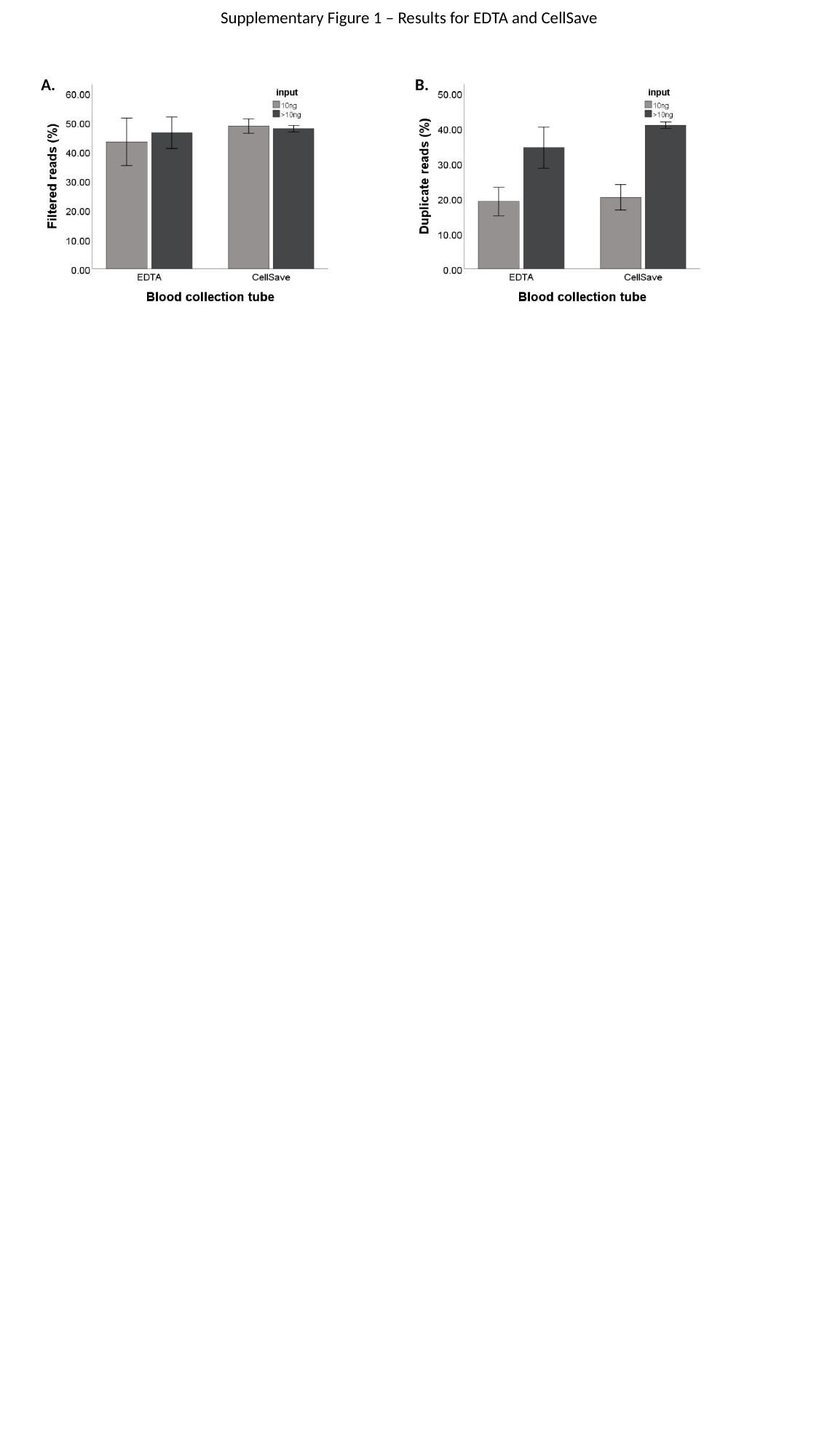

Supplementary Figure 1 – Results for EDTA and CellSave
A.
B.

Supplement: Supplementary file 1 — Additional file 1: Figure S1. The compatibility of different blood collection tubes with MeD-seq analysis. Percentages of A. LpnPI filtered reads and B. duplicate reads are shown for EDTA and CellSave tubes obtained during the same blood draw from 3 patients. For each sample, 10 ng cfDNA and maximal cfDNA input in 8 µl (> 10 ng) was analysed, in which the maximal amount was kept equal between EDTA and CellSave per patient. [file 13148_2021_1177_MOESM1_ESM.pptx]

## Slide 1
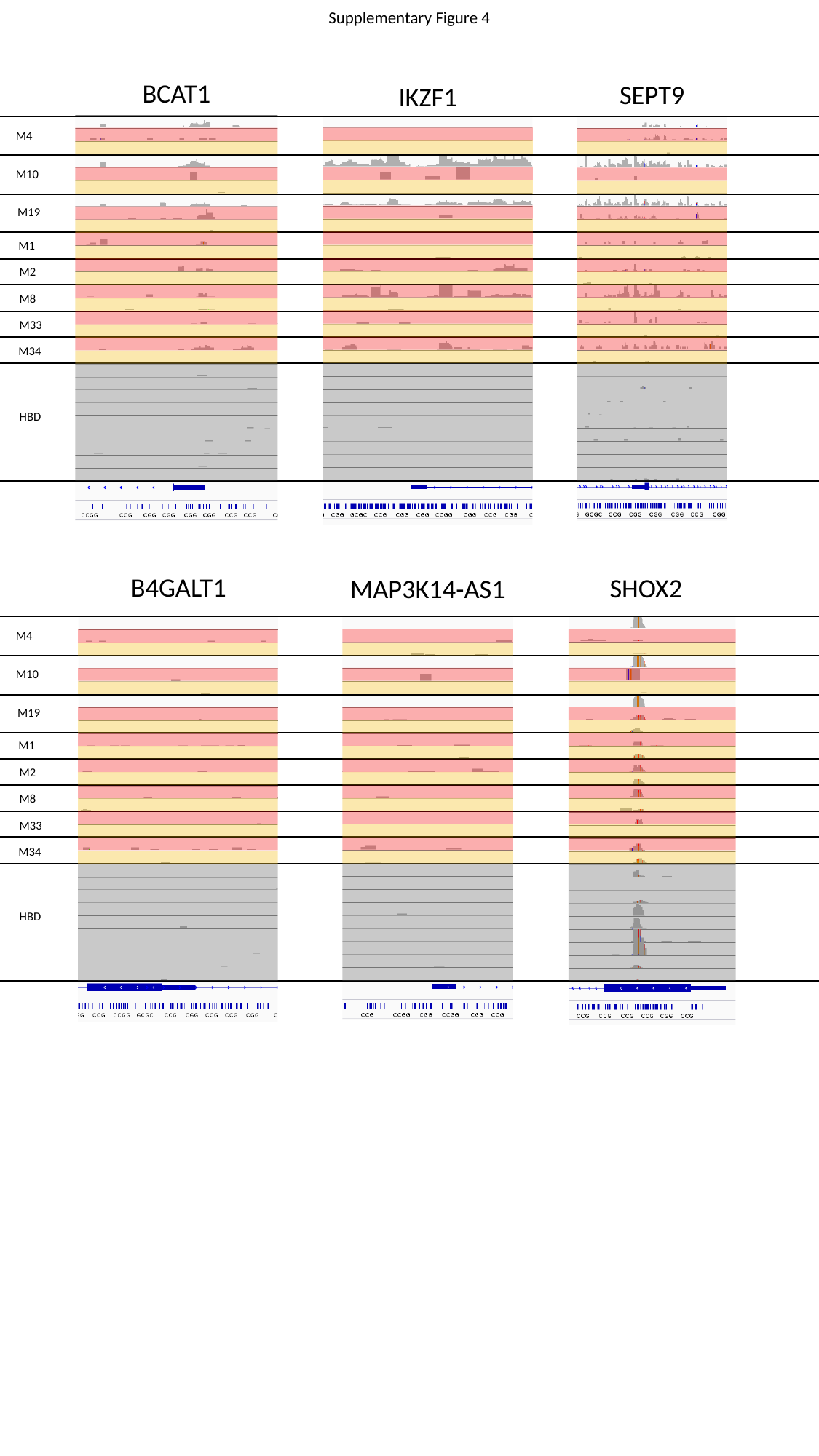

Supplementary Figure 4
BCAT1
SEPT9
IKZF1
M4
M10
M19
M1
M2
M8
M33
M34
HBD
B4GALT1
SHOX2
MAP3K14-AS1
M4
M10
M19
M1
M2
M8
M33
M34
HBD

Supplement: Supplementary file 4 — Additional file 4: Figure S4. MeD-seq results for published biomarkers on cfDNA from HBDs and paired tissue, pre-operative and post-operative cfDNA from CRLM patients. Sequenced MeD-seq reads shown for BCAT1, IKZF1, SEPT9, B4GALT1, MAP3K14-AS1, and SHOX2 from pre-operative cfDNA (red) and post-operative cfDNA (orange) of CRLM-patients and cfDNA samples of HBDs (black). Results are visualized in Integrative Genomics Viewer (IGV) v.2.9.4. [file 13148_2021_1177_MOESM4_ESM.pptx]

## Slide 1
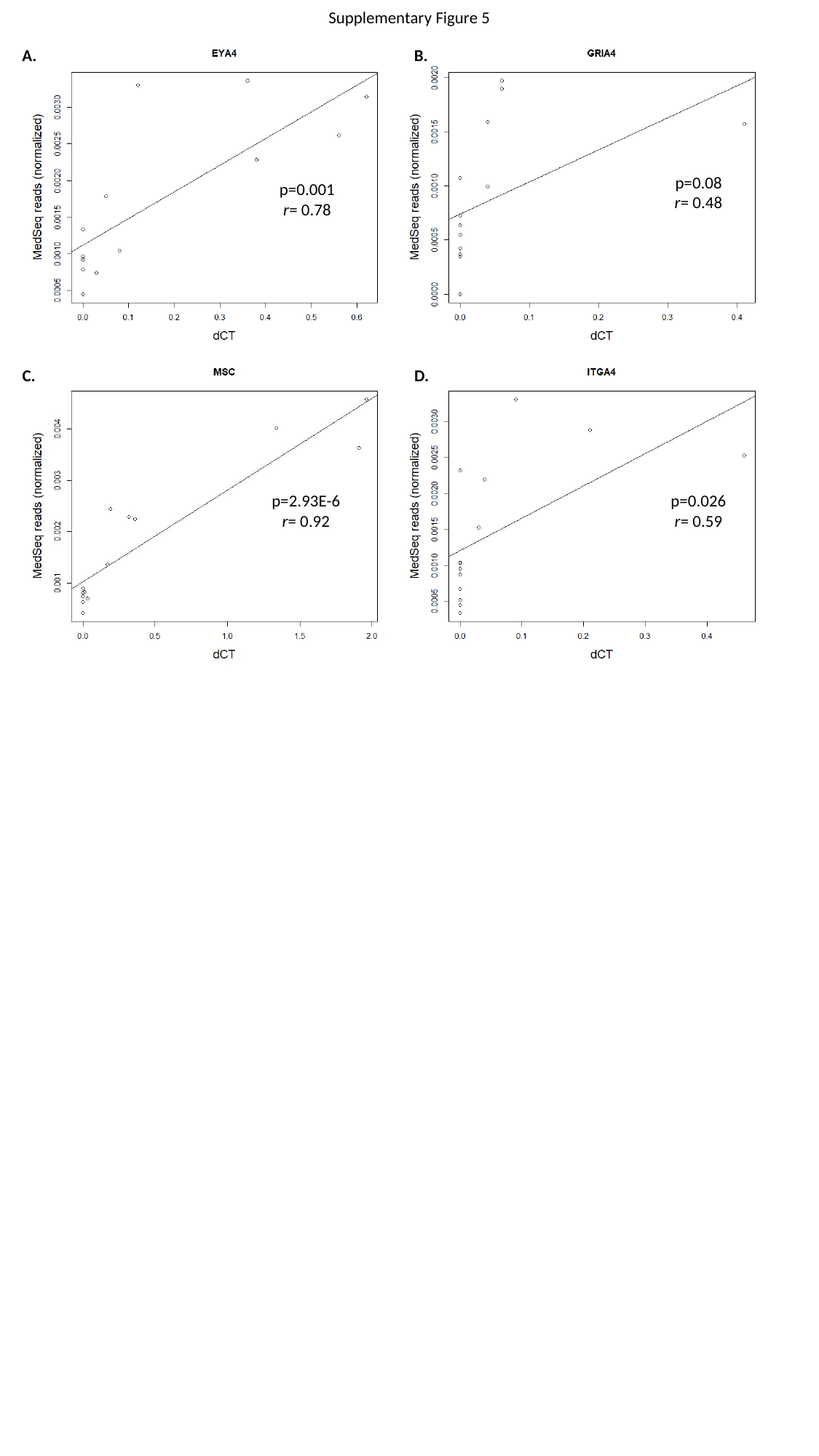

Supplementary Figure 5
A.
B.
p=0.08
r= 0.48
p=0.001
r= 0.78
C.
D.
p=0.026
r= 0.59
p=2.93E-6
r= 0.92

Supplement: Supplementary file 5 — Additional file 5: Figure S5. Correlation between MeD-seq and qMSP for previously identified markers for CRLM. Scatterplots showing the Pearson correlations between MeD-seq and qMSP results for A. EYA4, B. GRIA4, C. MSC, and D. ITGA4. [file 13148_2021_1177_MOESM5_ESM.pptx]
