# Supplementary material for: High-throughput and affordable genome-wide methylation profiling of circulating cell-free DNA by methylated DNA sequencing (MeD-seq) of LpnPI digested fragments
Source: Clin Epigenetics. 2021 Oct 20;13:196. doi: 10.1186/s13148-021-01177-4 (PMC8529776; doi:10.1186/s13148-021-01177-4)
Supplement: Supplementary file 2 — Additional file 2: Figure S2. Observed correlations between biological replicates compared to HBDs. Boxplots show significant higher Pearson’s r between biological replicates per patient (n = 4), in white, compared to the Pearson’s r between these samples and unrelated HBD samples (n = 9), in grey (Mann–Whitney U, p = 0.015 for M4, p = 0.004 for M10, and p < 0.001 for M19). Biological duplicates were taken during the same blood draw using either EDTA or CellSave tubes, of which either 10 ng or the maximum yield in 8 µl was used for MeD-seq. [file 13148_2021_1177_MOESM2_ESM.pptx]

## Slide 1
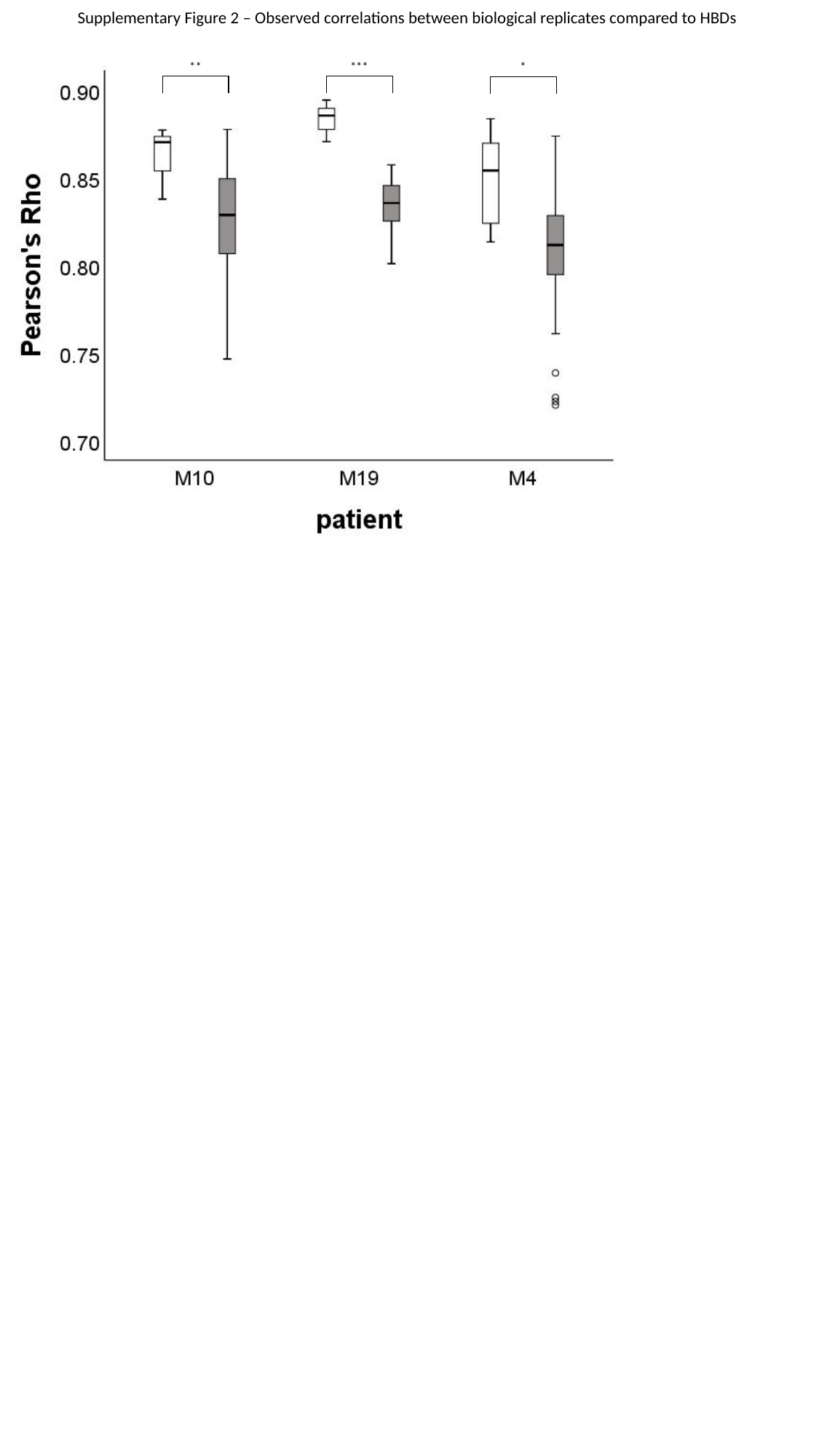

Supplementary Figure 2 – Observed correlations between biological replicates compared to HBDs
**
***
*
