# Supplementary material for: High-throughput and affordable genome-wide methylation profiling of circulating cell-free DNA by methylated DNA sequencing (MeD-seq) of LpnPI digested fragments
Source: Clin Epigenetics. 2021 Oct 20;13:196. doi: 10.1186/s13148-021-01177-4 (PMC8529776; doi:10.1186/s13148-021-01177-4)
Supplement: Supplementary file 3 — Additional file 3: Figure S3. Leukocyte-specific markers in HBDs. A heat map of a subset of leukocyte-specific methylation markers described by Accomando et al. (17) shows consistent methylation patterns across the included healthy blood donors. Only regions showing at least 1 read in 9 HBD samples are included. [file 13148_2021_1177_MOESM3_ESM.pptx]

## Slide 1
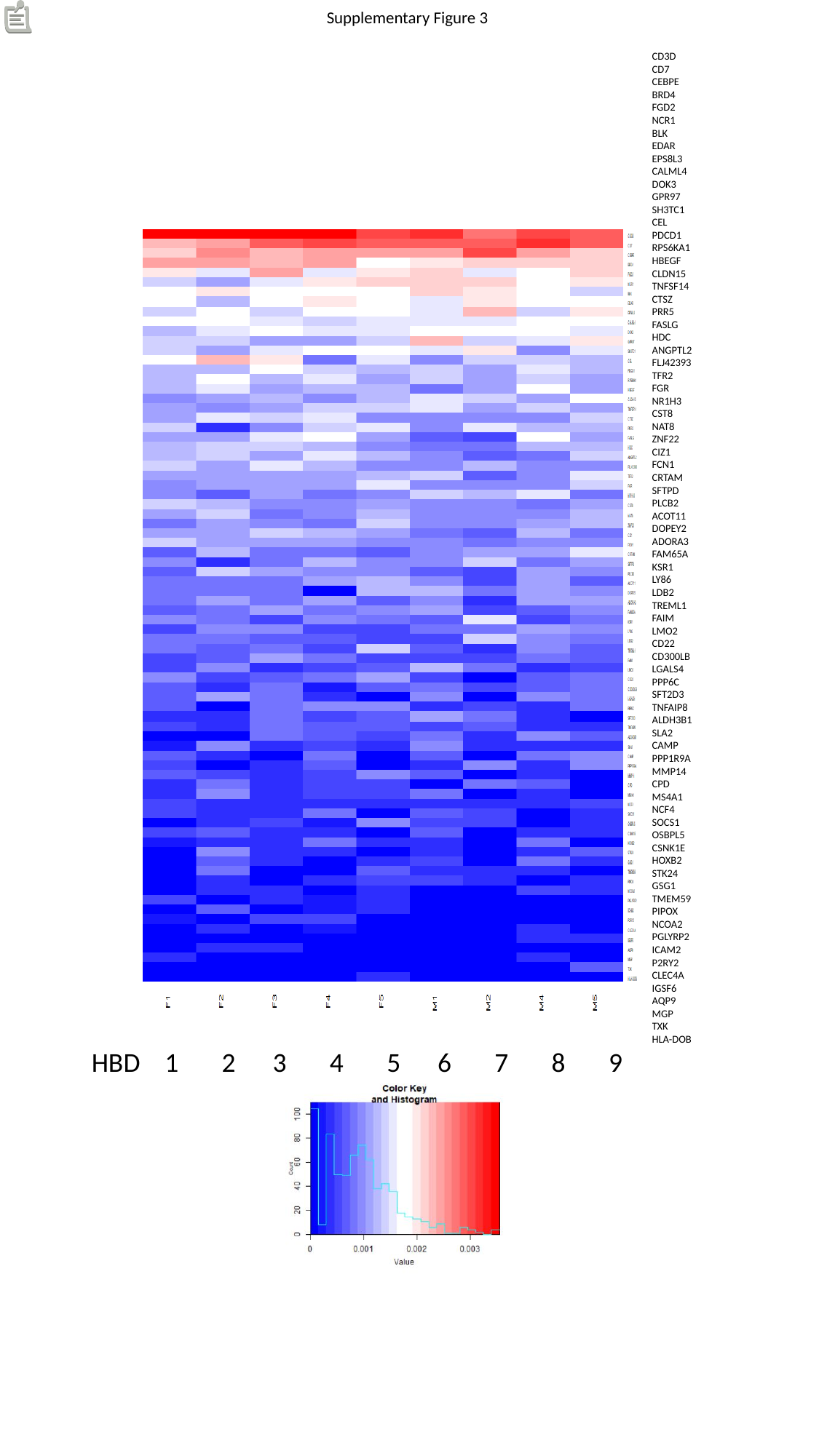

Supplementary Figure 3
| CD3D |
| --- |
| CD7 |
| CEBPE |
| BRD4 |
| FGD2 |
| NCR1 |
| BLK |
| EDAR |
| EPS8L3 |
| CALML4 |
| DOK3 |
| GPR97 |
| SH3TC1 |
| CEL |
| PDCD1 |
| RPS6KA1 |
| HBEGF |
| CLDN15 |
| TNFSF14 |
| CTSZ |
| PRR5 |
| FASLG |
| HDC |
| ANGPTL2 |
| FLJ42393 |
| TFR2 |
| FGR |
| NR1H3 |
| CST8 |
| NAT8 |
| ZNF22 |
| CIZ1 |
| FCN1 |
| CRTAM |
| SFTPD |
| PLCB2 |
| ACOT11 |
| DOPEY2 |
| ADORA3 |
| FAM65A |
| KSR1 |
| LY86 |
| LDB2 |
| TREML1 |
| FAIM |
| LMO2 |
| CD22 |
| CD300LB |
| LGALS4 |
| PPP6C |
| SFT2D3 |
| TNFAIP8 |
| ALDH3B1 |
| SLA2 |
| CAMP |
| PPP1R9A |
| MMP14 |
| CPD |
| MS4A1 |
| NCF4 |
| SOCS1 |
| OSBPL5 |
| CSNK1E |
| HOXB2 |
| STK24 |
| GSG1 |
| TMEM59 |
| PIPOX |
| NCOA2 |
| PGLYRP2 |
| ICAM2 |
| P2RY2 |
| CLEC4A |
| IGSF6 |
| AQP9 |
| MGP |
| TXK |
| HLA-DOB |
HBD 1 2 3 4 5 6 7 8 9
